# Link Prediction in Complex Networks Using Average Centrality-Based Similarity Score

**DOI:** 10.3390/e26060433

**Published:** 2024-05-21

**Authors:** Y. V. Nandini, T. Jaya Lakshmi, Murali Krishna Enduri, Hemlata Sharma

**Affiliations:** 1 Algorithms and Complexity Theory Lab, Department of Computer Science and Engineering, SRM University-Andhra Pradesh, Amaravati 522502, India; nandini_y@srmap.edu.in (Y.V.N.); muralikrishna.e@srmap.edu.in (M.K.E.); 2 Department of Computing, Sheffield Hallam University, Sheffield S1 2NU, UK

**Keywords:** complex networks, link prediction measures, centrality measures

## Abstract

Link prediction plays a crucial role in identifying future connections within complex networks, facilitating the analysis of network evolution across various domains such as biological networks, social networks, recommender systems, and more. Researchers have proposed various centrality measures, such as degree, clustering coefficient, betweenness, and closeness centralities, to compute similarity scores for predicting links in these networks. These centrality measures leverage both the local and global information of nodes within the network. In this study, we present a novel approach to link prediction using similarity score by utilizing average centrality measures based on local and global centralities, namely Similarity based on Average Degree $\left(SAC_{D}\right)$, Similarity based on Average Betweenness $\left(SAC_{B}\right)$, Similarity based on Average Closeness $\left(SAC_{C}\right)$, and Similarity based on Average Clustering Coefficient $\left(SAC_{CC}\right)$. Our approach involved determining centrality scores for each node, calculating the average centrality for the entire graph, and deriving similarity scores through common neighbors. We then applied centrality scores to these common neighbors and identified nodes with above average centrality. To evaluate our approach, we compared proposed measures with existing local similarity-based link prediction measures, including common neighbors, the Jaccard coefficient, Adamic–Adar, resource allocation, preferential attachment, as well as recent measures like common neighbor and the Centrality-based Parameterized Algorithm (CCPA), and keyword network link prediction (KNLP). We conducted experiments on four real-world datasets. The proposed similarity scores based on average centralities demonstrate significant improvements. We observed an average enhancement of 24% in terms of Area Under the Receiver Operating Characteristic (AUROC) compared to existing local similarity measures, and a 31% improvement over recent measures. Furthermore, we witnessed an average improvement of 49% and 51% in the Area Under Precision-Recall (AUPR) compared to existing and recent measures. Our comprehensive experiments highlight the superior performance of the proposed method.

## 1. Introduction

A graph is used to represent a complex network, where nodes or vertices represent entities, and edges or links represent the interactions or relations between these entities. Complex networks play a major role in natural phenomena, including biological networks, information networks, social networks, and technological networks [1,2,3]. In such networks, nodes are neurons, scientists, individuals, or locations, whereas edges are associations or interactions between the nodes. In recent times, complex networks have gained significant attention in various fields including link prediction [4], centrality measures [5], community detection [6], and influence maximization [7]. New nodes and links are constantly added to complex networks, which makes these networks dynamic. The challenge of predicting links in a network is therefore critical to comprehend the network’s evolution. The link prediction (LP) problem was introduced by Liben-Nowell et al. [8]. The LP problem aims to determine the probability of an interaction happening in the future between two nodes when such an interaction does not exist at a present moment in time. There is potential significance for the link prediction problem across multiple domains. Techniques for link prediction can be utilized to determine the interactions in biological networks that are the most likely to occur, thereby considerably reducing the costs associated with conducting experiments. Link prediction can be used to send friend requests on social networks such as Facebook and LinkedIn. On e-commerce platforms like Amazon, users can receive product recommendations by predicting connections between users and items. This is done using a user–item graph that represents user preferences or purchase history. Link prediction in co-authorship networks such as DBLP might point to possible partnerships between researchers [9]. Numerous link prediction algorithms have been proposed recently. These algorithms are classified into three groups: similarity-based measures [10,11], probabilistic measures [12], and dimensionality-based measures [13]. In particular, the most efficient and fundamental techniques for resolving the link prediction problem is the similarity-based measure. This approach computes a score, $S_{v,u}$, for each pair of nodes (v,u), that indicates how similar the two nodes are to one another. Two nodes are considered similar if they share a large number of features, according to the general definition. The similarity indices are divided into three groups: local, global, and quasi-local [14]. In order to compute a node’s similarity, local similarity indices employ structural information from their neighbors rather than the entire network. A few popular local similarity measures are common neighbors, the Jaccard coefficient, preferential attachment, resource allocation, and Adamic–Adar. These measures are discussed in Section 4.1. In this study, we defined a new similarity measure that belongs to the class of common neighbor measures [15] and we used this as a basis for link prediction. These measures evaluate the probability of a link forming between non-adjacent pairs of nodes in a network based on the quantity of common neighbors they share. The primary drawback of local similarity indices is their limited ability to utilize local data; they use only one-hop and two-hop neighborhoods [16]. However, links can emerge between nodes existing beyond two-hop neighborhood. Global similarity indices utilize the entire network’s structural information to evaluate link scores. However, they are not parallelizable and their computational complexity limits efficiency in large networks. Conversely, quasi-local similarity indices combine the best features of both methods. In order to retain accuracy, quasi-local indices use more information than local indices and omit unnecessary information [17]. The use of centrality-based link prediction has several advantages over traditional methods. Firstly, centrality measures help analysts evaluate the relative importance of nodes and edges in the network, which is crucial for predicting new connections. This leads to a more detailed understanding of the network’s structure and dynamics, enabling more precise and informed predictions. Moreover, centrality measures, such as the clustering coefficient, measure the extent to which nodes in a network tend to form clusters, whereas closeness centrality is found to better describe endpoint influence, and betweenness centrality best quantifies path connectivity. This comprehensive assessment of a node’s influence and importance within the network leads to more accurate predictions of future links.

This paper’s outline is structured as follows: Section 2 and Section 3 describe problem definitions and recent works on link prediction and centrality measures. Section 4 discusses the related existing measures. Section 5 presents the methodology, including the centrality measures utilized, the calculation of average centrality, and the definition of similarity scores. Section 6 describes the experimental setup and presents the evaluation results. Section 7 provides an in-depth analysis and comparison with existing measures and recent measures. Section 8 concludes the paper and outlines potential directions for future research. Finally, Abbreviations defines the abbreviations used in this paper.

## 2. Problem Definition

**Definition 1.**  *Link Prediction: The link prediction task involves a complex network denoted as G=(V,E), where V represents the set of vertices and E represents the set of edges. The objective is to generate a list of edges that are not currently present in the network $G\left[t_{0},t_{i}\right]$, but are predicted to appear in the future network $G\left[t_{j}\right]$ where $t_{j}>t_{i}>t_{0}$ [4].*

The graph *G* may include directed edges indicating one-way interactions between nodes, along with weights indicating the strength of these interactions. However, this study focuses solely on undirected and unweighted edges. The potential expansion of this research to include directed and weighted networks is a prospect for future work.

**Definition 2.**  *Centrality Measure: Given a graph G=(V,E), where V and E denote vertex and edge sets, respectively, the centrality, represented as C and defined as C:V→R, assigns a real-valued score to u, quantifying the significance of u based on its structural position and connections to other nodes in G.*

Various centrality measures exist, each capturing different aspects of a node’s importance. Common centrality metrics include degree centrality, which measures the number of connections a node has, and betweenness centrality, which quantifies how often a node lies on the shortest paths between other nodes in the graph. Other measures include closeness centrality and the clustering coefficient, each providing unique insights into a node’s centrality within the network.

The formation of future links in a network between two non-adjacent nodes *u* and *v* majorly depends on the structural similarity of *u* and *v*. A key factor influencing this resemblance is the presence of shared neighbors between *u* and *v*. However, many existing methods for predicting links fail to differentiate between these common neighbors. We believe that all common neighbors may not contribute equally in future link formation. In this work, we intend to evaluate the role of significance of common neighbors in link prediction. As the centrality of nodes depict different kinds of significance in the network, the centrality value of common neighbors affect link formation. Therefore, in this work, we examine various centrality values of nodes (especially common neighbors) on the task of link prediction.

## 3. Recent Work

This section addresses the latest research on link prediction using centrality measures. Lu et al. [15] summarized recent works on link prediction algorithms, and also introduced some real-time applications, as well as outlined the upcoming challenges of link prediction algorithms. Das et al. [18] presented research works on centrality measures based on social networks. The authors presented real-time applications of centrality measures in traffic, biology, transportation, research, drugs, and security. Bloch et al. [19] discussed centrality measures in networks based on nodal statistics and also discussed some properties which identify path-based centrality measures. Nasiri et al. [20] proposed new link prediction measures, namely weighted common neighbors (WCNs), depending on common neighbors and different types of centrality measures like degree, closeness, betweenness, k-core, eigenvector, and pagerank, which are used to predict the formation of new links in networks. To measure the performance of centrality measures based on link prediction, Singh et al. [21] investigated centrality measure network structures, then identified influential users and predicted future connections. Ahmad et al. [22] proposed a novel measure, called common neighbor and the Centrality-based Parameterized Algorithm (CCPA), which is parameterized and identifies future edges between non-adjacent node pairs using common neighbors and centralities. The next novel measure called the keyword network link prediction algorithm (KNLP) was proposed by Behrouzi et al. [23], which exploits nodes’ clustering coefficient, centrality measures using eigenvector centrality, and community information, which can be used as an another parameter to predict the links based on centrality values. S Kumar et al. [24] proposed link prediction based on centralities of nodes, which improves the set of features that are utilized to make the predictions. The basic node centralities and various binary machine learning classifiers are used to predict links. T Gao et al.’s [25] focus was on degrees of end points and neighbors, so the authors proposed a powerful combination of endpoints and neighbors (PCEN) model, which gets better prediction results than existing models. Kumar et al. [26] proposed a new approach to link prediction based on the level-2 node clustering coefficient. To compute similarity scores between node pairs, the authors defined level-2 common nodes and their clustering coefficient, which extracts level-2 common neighbors’ clustering information from the seed node pairs. Based on the rich get richer scenario, Zhang et al. [27] proposed an novel index relying on betweenness centrality to predict the links that will exist in the future. Later, Wu et al. [28] proposed local triangle structure information, which can be transformed by the clustering coefficient of common neighbors directly. Yang et al. [29] proposed an algorithm, named common neighbors and distance which excels in predicting missing links between nodes without common neighbors, outperforming many existing methods for real-world networks without adding any complexity. In this paper, we generalized similarity scores based on average centrality measures, which were calculated using local and global centrality measures, which give the best prediction accuracy compared to existing link prediction measures.

## 4. Related Work

In this section, we discuss basic link prediction and centrality measures for simple, unweighted, and undirected graphs. G=(V,E) is a representation of a network or graph, where *V* is the number of nodes and *E* is the collection of network edges.

### 4.1. Existing Similarity Measures

A straightforward method, known as “similarity-based method”, computes a similarity score for non-adjacent node pairs, *v* and *u*. The similarity scores are sorted; the node pairs with the highest scores indicate the expected linkages between them. Similarity scores are grouped into local, global, and quasi-local groups [4].

- **Local Similarity Measures:** Local similarity measures focus on examining the immediate neighbors of a node in the network. Some well-known measures include the common neighbor (CN) [15], Jaccard coefficient (JC) [3], preferential attachment (PA) [30], Adamic–Adar (AA) [31], resource allocation (RA) [32], etc.
  **Common Neighbor:** The likelihood of a link being formed between two nodes, *v* and *u*, is higher when they share a significant number of common neighbors.
  (1) $$S_{v,u}^{CN}=\left|\Gamma \left(v\right)\cap \Gamma \left(u\right)\right|$$
  In Equation (1), $S_{v,u}^{CN}$ denotes the size of the nodes’ neighborhoods’ intersection; Γ(v) is the set of neighbors of node *v*.
  **Jaccard Coefficient:** The common neighbor is comparable to this metric, which normalizes the score of the common neighbor, as given below.
  (2) $$S_{v,u}^{JC}=\frac{\left|\Gamma (v)\cap \Gamma (u)\right|}{\left|\Gamma (v)\cup \Gamma (u)\right|}$$
  In Equation (2), $S_{v,u}^{JC}$ is the size of the intersection of two nodes’ neighborhoods, out of the total neighbors of nodes *v* and *u*, where Γ(v) is the set of neighbors of node *v*.
  **Preferential Attachment:** It counts the richness of two nodes instead of shared neighbors between non-adjacent node pairs. The degrees of nodes *v* and *u* are multiplied collectively.
  (3) $$S_{v,u}^{PA}=\left|d\left(v\right)|*|d\left(u\right)\right|$$
  PA requires the degree of nodes and does not consider common neighbors. In Equation (3), d(v) is the degree of node *v*.
  **Resource Allocation:** We assume two non-adjacent node pairs, *v* and *u*. The amount of resources provided from node *v* to node *u* determines how similar the two nodes are when they are transferring resources through their shared nodes.
  (4) $$S_{v,u}^{RA}=\sum_{r\in \Gamma (v)\cap \Gamma (u)}\frac{1}{d_{r}}$$
  In Equation (4), $d_{r}$ is the degree of node *r*.
  **Adamic–Adar:** Adamic–Adar is a variant of resource allocation. In real-world scenarios, for example, individuals with a larger number of friends tend to allocate less time and resources to particular friend compared to those with fewer friends. This is defined as follows:
  (5) $$S_{v,u}^{AA}=\sum_{r\in \Gamma (v)\cap \Gamma (u)}\frac{1}{log|d_{r}|}$$
  In Equation (5), $d_{r}$ is the degree of node *r*.

### 4.2. Recent Measures

In this section, two of recent centrality based similarity scores: CCPA [22] and KNLP [23] are elaborated.

- **Common Neighbor and Centrality-based Parameterized Algorithm (CCPA):** To recommend the creation of new linkages in complex networks, CCPA uses two essential node characteristics—the number of shared neighbors between node pairs, and their centrality measures. In this case, closeness centrality is taken into account as a parameter for missing link prediction. The term “common neighbor” describes the nodes that are shared by two nodes. The term “centrality” refers to the significance of a node inside the network.
  (6) $$S_{v,u}^{CCPA}=\alpha \cdot \left(|\Gamma (v)\cap \Gamma (u)|\right)+\left(1-\alpha \right)\cdot \frac{N}{D_{v,u}}$$
  In Equation (6), the user-generated parameter α∈[0,1] regulates the centrality and common neighbor relevance. The set of neighbors of node *v* is represented by Γ(v), and $D_{v,u}$ is the shortest path length between *v* and *u*.
- **Keyword Network Link Prediction Algorithm (KNLP):** KNLP depends on the nodes’ clustering coefficient, and their centrality measure like eigenvector centrality [33]. The stronger correlation between eigenvector centrality and node degree shows that nodes with the highest eigenvector have more connections. For nodes *u* and *v*, KNLP is defined as follows:
  (7) $$S_{v,u}^{KNLP}=\frac{CS_{v}+CS_{u}}{CC_{v}+CC_{u}+\epsilon }$$
  In Equation (7), $CS_{v}$ and $CS_{u}$ are the centrality scores for nodes *v* and *u*, $CC_{v}$ and $CC_{u}$ are clustering coefficient values for nodes *v* and *u*, and their values always range between 0 and 1. Here, ϵ is used to avoid the division by zero error.

### 4.3. Centrality Measures

Centrality measures identify the nodes that are most crucial or central in the graph *G*. These measures help us to understand which nodes are the most influential, well-connected, or central in the graph. Centralities are derived into local measures, global measures, and so on [34].

- **Local Centrality:** Local centrality involves only immediate neighborhood. Degree centrality (D) [5] and clustering coefficient (CC) [35] are two popular local centralities used in this paper.
  **Degree Centrality:** The node *v*’s degree centrality is calculated as the fraction of other nodes adjacent to node *v* out of the possible total. Nodes characterized by a high degree of centrality are referred to as Hub nodes.
  (8) $$C_{D}\left(v\right)=\frac{d_{v}}{N-1}$$
  In Equation (8), the graph’s total number of nodes is *N*, and node *v* has a degree of $d_{v}$.
  **Clustering Coefficient:** The clustering coefficient of a specific node is determined by the ratio of closed triangles within the node’s neighborhood, to the total number of triangles present in that neighborhood. It is also known as transitivity.
  (9) $$C_{CC}\left(v\right)=\frac{2K_{v}}{d_{v}\left(d_{v}-1\right)}$$
  In Equation (9), node *v* has a degree of $d_{v}$, and the number of triangles connected to node *v* is $K_{v}$.
- **Global Centrality:** Global centrality involves the whole graph. Closeness centrality (C) [34] and betweenness centrality (B) [36] are few popular global centralities used in this paper.
  **Closeness Centrality:** One method of identifying nodes that can efficiently distribute information throughout a network is through closeness centrality. The closeness centrality of a node, denoted as *v*, within a graph, is determined by taking the reciprocal of the average shortest path distance from node *v* to all N−1 reachable nodes in the graph.
  (10) $$C_{C}\left(v\right)=\frac{N-1}{\sum_{u\neg v}D_{v,u}}$$
  In Equation (10), the shortest path length from *v* to *u* is denoted by $D_{v,u}$. In the network, the node that is nearest to every other node is the one with the highest closeness centrality.
  **Betweenness Centrality:** A node’s betweenness centrality is a measure of how many shortest paths there are via a particular node.
  (11) $$C_{B}\left(v\right)=\sum_{v,u\in V}\frac{\sigma_{v,u}\left(r\right)}{\sigma_{v,u}}$$
  In Equation (11), $\sigma_{v,u}$ represents the total number of shortest paths between nodes *v* and *u*, and $\sigma_{v,u}\left(r\right)$ denotes the total number of shortest paths between nodes *v* and *u* that pass through node *r*.

## 5. Proposed Work

In this section, we outline our proposed approach for predicting links, which relies on the average centrality of the common neighbors. The proposed method computes a prediction score based on similarity between the nodes, which is based on the centrality score of the common neighbors between them. We name this method Similarity based on Average Centrality (SAC). SAC initially computes various centrality scores for the common neighbors and considers only the nodes with scores exceeding the network’s overall average centrality score. We employ both local and global centrality measures.

### 5.1. Similarity Based on Average Centrality Measures (SAC)

The algorithm SAC can be generalized to use any centrality measure of nodes. Let C denote the centrality score of a node *v* and *A*C(*G*) denote a graph’s average centrality value computed using Equation (12).

(12) $$AC\left(G\right)=\frac{\sum_{v\in V(G)}C\left(v\right)}{N}$$

In Equation (12), C(v) represents the centrality value of the node *v*, and *N* denotes the total number of nodes in the whole graph *G*. The similarity of two vertices using the average centrality of a graph is defined as depicted in Equation (13):

(13) $$SAC_{C}\left(v,u\right)=|\left\{x|x\in \Gamma \left(v\right)\cap \Gamma \left(u\right)\;\mathrm{and}\;C\left(x\right)\geq AC\left(G\right)\right\}|$$

In Equation (13), $SAC_{C}\left(v,u\right)$ is the similarity scores of node pairs *v* and *u*, collecting all common neighbors and applying centrality scores to those common neighbors and then counting the nodes which exceed the average centrality of the graph. *x* denotes common neighbors between nodes *v* and *u*, and Γ(v) and Γ(u) are neighbors of the nodes *v* and *u*, respectively. AC is the average centrality of the graph, which is defined in Equation (12). The centrality C can be any local or global centralities defined in Table 1.

For instance, if we consider the centrality C to denote the degree centrality, we can utilize the average degree centrality (AD) as defined in Equation (12). This enables us to calculate the similarity between two vertices based on the average degree centrality of the graph, as specified in row 1 of Table 1. C can be tailored to the betweenness centrality, closeness centrality, or clustering coefficient by using the second, third, and fourth rows of Table 1, respectively, leading to the computation of $SAC_{B}\left(v,u\right)$, $SAC_{C}\left(v,u\right)$, and $SAC_{CC}\left(v,u\right)$.

Algorithm 1 outlines the process for calculating the $SAC_{C}\left(v,u\right)$ for non-adjacent node pairs within the graph.
**Algorithm 1:** An algorithm for common neighbor-based average centrality
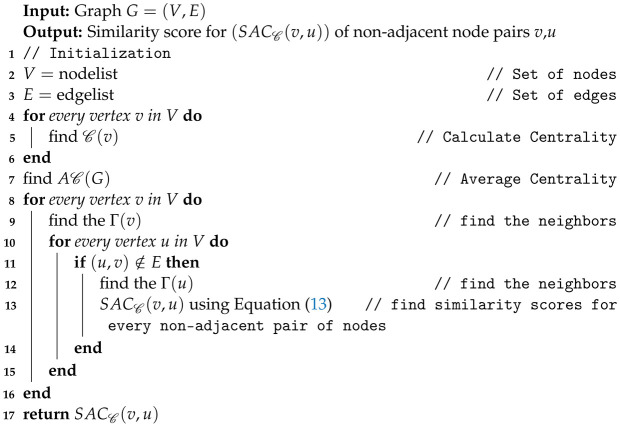


**Figure 1:**
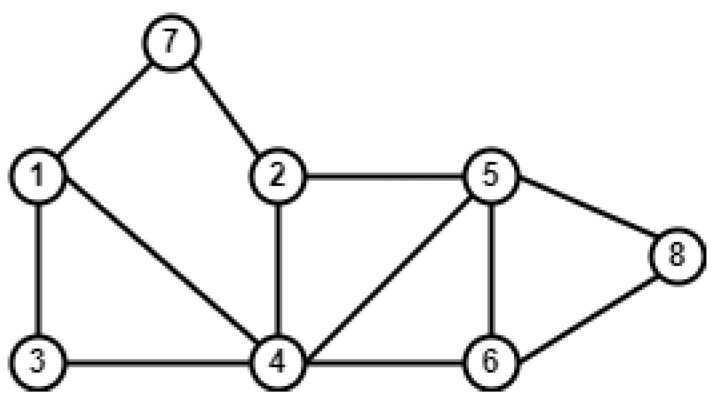
An illustration of an undirected toy network with eight nodes and twelve edges.

A sample illustration of Algorithm 1 is given using a toy example, depicted in Figure 1, featuring eight nodes and twelve edges.

For the graph given in Figure 1, we find similarity scores for $SAC_{D}\left(v,u\right)$, $SAC_{B}\left(v,u\right)$, $SAC_{C}\left(v,u\right)$, $SAC_{CC}\left(v,u\right)$, CN,JC,AA,RA,PA,CCPA, and KNLP. In this example, we find similarity scores for few non-adjacent node pairs; similarly, we can find similarity scores for other non-adjacent node pairs as well. We present the computation of similarity scores using the average centrality measure, with the degree centrality C being our chosen metric.

Initially, we calculate the degree centrality for each node in the graph. Node 1 and Node 2, for instance, both exhibit a degree centrality of 0.375, and so forth. Subsequently, we determine the average degree centrality for the graph, denoted as AD(G), as specified in Table 1, line 1. For our toy graph, AD(G) equals 0.375. Next, to identify common neighbors for a non-adjacent node pair (1,2), we locate Nodes 4 and 7. Applying the degree centrality scores to these common neighbors, we find that Node 4 has a centrality of 0.625, and Node 7 has a centrality of 0.25. Finally, we count the nodes with centrality scores exceeding the average degree centrality. In this scenario, the common neighbor Node 4 surpasses the average degree centrality. Consequently, the similarity between node pairs (1,2), based on average degree centrality, is 1. This process is repeated for several node pairs in the toy graph, and the results are summarized in Table 2.

### 5.2. Time Complexity of Similarity Based on Average Centrality Measures

Given the network G=(V,E), where the number of nodes is indicated by |V|=n, and the number of edges is represented by |E|=m, the computational cost of evaluating the C for every vertex in a graph *G* can be expressed as O(f(n)). The complexity for finding the similarity score $SAC_{C}$ in Algorithm 1 is $O(f\left(n\right)+O\left(n^{2}\right))$. In the case where C is the degree, the time complexity for finding the $SAC_{D}$ is $O(n^{2})$ [5,37]. If the C is the clustering coefficient, betweenness centrality, and closeness centrality, then the time complexity for finding the $SAC_{B}$, $SAC_{C}$, and $SAC_{CC}$ is O(nm) [34,35,36].

## 6. Implementation

The proposed approach’s effectiveness was compared to a few popular cutting-edge link prediction measures. The datasets utilized for performance analysis and the measures used for evaluation are described in depth in this section.

### 6.1. Datasets

To evaluate the effectiveness of our proposed method, we conducted simulations on four different datasets. These datasets were taken from different domains and were downloaded from [38]. In bio-celegans, nodes represent genes or proteins, where edges are interactions between the proteins. The dataset comprises a total of 453 nodes and 2025 edges. The web-polblogs dataset represents a network of political blogs, where webpages are the nodes and hyperlinks between webpages are the edges. It consists of 643 nodes and 2280 edges. The CA-Grqc dataset represents a collaboration network, where nodes are authors or research papers and edges represent relationships between authors or citations between research papers. It consists of 5242 nodes and 14,496 edges. The last dataset used was Facebook-large dataset, which represents a social network, where nodes specify users and the edges represent friendship between users. It consists of 22,470 nodes and 171,002 edges. Table 3 displays the characteristics of these datasets. Among all of these datasets, bio-celegans is a dense network with a relatively high average clustering coefficient. CA-Grqc has a low average degree, which indicates a lower average number of connections per node. Facebook-large has high average degree, which indicates a well-connected network and it has a relatively low diameter, suggesting shorter paths between nodes compared to CA-Grqc.

Our study was carried out using a PC with an 11th generation Intel(R) with Core(TM) i7-8700 CPU, which has six cores, twelve logical processors, and a base clock speed of 3.20 GHz. The computer was running Windows 10 Education and had 16 GB of RAM. Python was used to perform our investigation, and Scikit-Learn, Matplotlib, Pandas, Networkx, and Numpy were among the packages used to build the methods.

For each of these datasets, 20% of the links were set aside for testing purposes. Prediction scores were calculated for the remaining 80% of the links. Subsequently, the effectiveness of the predictions was assessed using both the Area Under the ROC curve and the Area Under the Precision-Recall curve. These evaluation metrics will be discussed further in the following section.

### 6.2. Evaluation Metrics

In the assessment of similarity-based centralities, standard metrics like Area Under the Receiver Operating Characteristic curve (AUROC) and Area Under the Precision-Recall curve (AUPR) are commonly employed. In our study, we employed these metrics to assess the performance of our proposed measures.

**AUROC:** AUROC, short for Area Under the Receiver Operating Characteristic (ROC), is a widely used metric for assessing the effectiveness of a prediction model. The ROC curve is a visual representation that illustrates the relationship between the True Positive Rate (TPR) and the (FPR). The TPR (y-axis) vs. FPR (x-axis) is plotted for various threshold values [39]. AUROC gives the area under the ROC curve. AUROC measures the probability of false alarms or incorrect positive predictions. The AUROC score has a range from 0 to 1, where a higher value signifies superior performance. An AUROC of 1 represents a perfect model, while an AUROC of 0.5 indicates a random model.

**AUPR:** AUPR stands for Area Under Precision-Recall (PR) curve, is another metric used to evaluate the performance of a prediction model. AUPR demonstrates superior performance in scenarios where the ROC curve may provide an overly optimistic assessment of a predictor’s performance, especially with imbalanced data [40,41]. The PR curve displays the precision on the y-axis and the recall on the x-axis. Precision quantifies the ratio of correct positive predictions to all positive predictions, while recall calculates the ratio of correct positive predictions to all actual positive instances. AUPR is a single quantity that represents the area under PR curve.

## 7. Results

In this section, we conducted experiments to evaluate the efficacy of the proposed approach. The obtained results are presented below for analysis. First, we compared our generalized SAC methods, proposed in Section 5, with existing local similarity measures like CN, JC, AA, RA, and PA and the latest link prediction measures, CCPA and KNLP, on four datasets. Our measures were tested on evaluation measures like AUROC and AUPR, as discussed in Section 6.2. We show that the performance of generalized SAC is good compared to existing link prediction measures. We have explained that the prediction of link score increases for SAC(v,u), by collecting all common neighbors for nodes *v*,*u* and applying centrality scores to those common neighbors and then counting the nodes which exceed the average centrality of the graph. In the section below, we discuss the results of the proposed algorithms based on popular existing link prediction measures, but we do not include the latest existing method, KNLP, in the table, as KNLP obtained comparatively small values. So, we present the results of KNLP separately in Table 4 and Table 5 for comparison.

### 7.1. Comparing Proposed Similarity-Based Centralities with Existing Similarity-Based Link Prediction Measures

**Figure 2:**
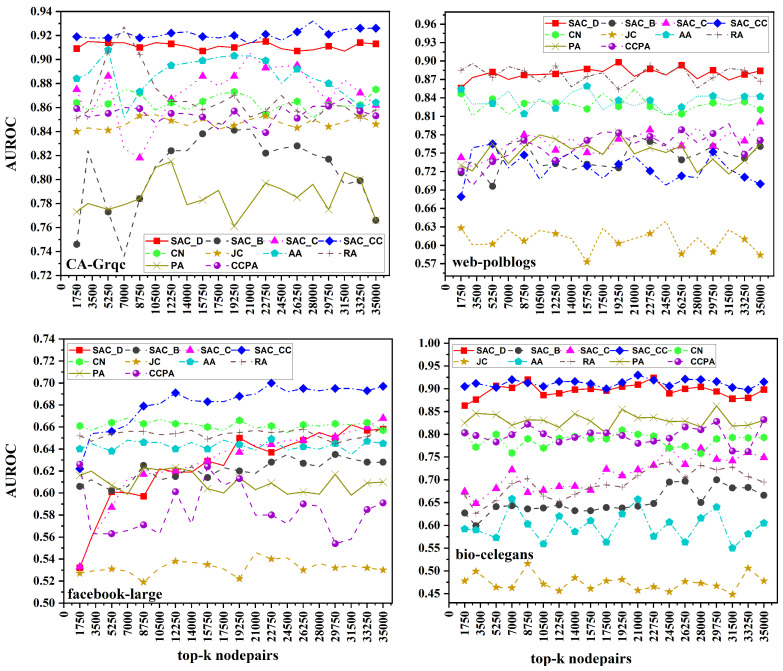
AUROC scores for link prediction using common neighbors based on average centrality for top *k* node pairs, *k* ranging from 1750 to 35,000, for four datasets.

The discussion about the results of the proposed generalized SAC measures is presented in this section. Average degree (AD), average betweenness (AB), average closeness (AC), and average clustering coefficient (ACC) are considered for the centrality C proposed in Section 5. These proposed measures are compared against the basic link prediction measures of CN, JC, AA, RA, PA, and CCPA. Figure 2 displays the AUROC findings for four datasets. While prediction scores are calculated for all non-adjacent node pairs, the evaluation is solely conducted on the top *k* pairs of nodes. This approach stems from the notion that node pairs with the highest scores are most likely to form connections in the future. We explored different values of *k* ranging from 1750 to 35,000. The AUROC and AUPR scores for *k* ranging from 1750 to 35,000 are given in Figure 2.

Let us choose a specific Facebook-large from the CA-Grqc dataset with *k* = 17,500 and the $SAC_{CC}$ measure where the AUROC is 0.918. This score suggests that, for this measure and dataset combination at this particular value of k, the model performed well in differentiating between positive and negative predictions in link prediction tasks. Essentially, the AUROC value of 0.918 indicates that there was a notable proportion of true positives compared to false positives across various threshold settings, resulting in this score.

In the CA-Grqc dataset, the proposed measure $SAC_{CC}$ on average demonstrated superior performance compared to all the baselines, followed by $SAC_{D}$, whereas the worst performing measure was PA, on average. The clustering patterns captured by $SAC_{CC}$ may provide more accurate predictions compared to the simplistic degree-based approach of preferential attachment, resulting in superior performance for $SAC_{CC}$. In the Facebook-large dataset, our measure $SAC_{CC}$ exhibited strong performance on average. In the Facebook dataset, $SAC_{CC}$ probably accounts for the network’s local clustering structure, meaning it does not only examine direct connections between nodes, but also relationships among their mutual friends. In contrast, traditional measures primarily concentrate on pairwise node relationships alone. In the web-polblogs dataset, our proposed $SAC_{D}$ and RA were comparable, as $SAC_{D}$ and RA focus on the number of neighbors a node has. In the bio-celegans dataset, $SAC_{D}$ obtained the highest scores in some k-node pairs, while $SAC_{CC}$ performed better in others. However, overall, $SAC_{CC}$ achieved the highest scores among all measures. In the Facebook-large, web-polblogs, and bio-celegans datasets, JC was the worst performing measure. This is because of the normalization of common neighbors, which tends to decrease the scores on large datasets with increasing numbers of nodes. Specifically, for CA-Grqc, our proposed measure $SAC_{CC}$ consistently outperforms AA by 5%, and CCPA, the latest measure, by 7%. For the Facebook-large dataset, the proposed $SAC_{CC}$ demonstrates a 0.9% enhancement compared to CN, and a significant 5% improvement over CCPA. In web-polblogs, $SAC_{CC}$ exhibits a competitive performance, outpacing RA by 0.3%, and surpassing CCPA, by 12%. Finally, for bio-celegans, $SAC_{CC}$ excels with an 8% improvement over PA and a notable 9% improvement over CCPA.

**Figure 3:**
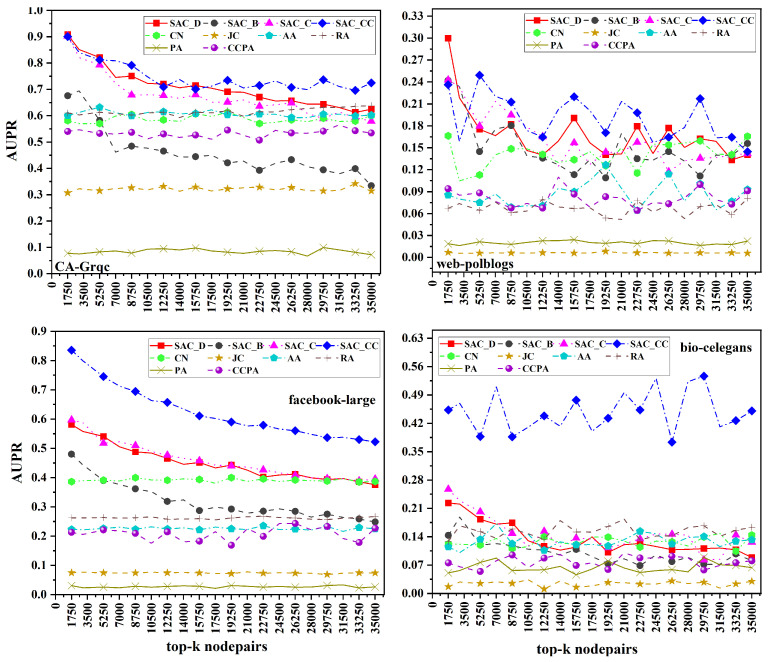
AUPR scores for link prediction using common neighbors based on average centrality for the top 35,000 node pairs across four datasets.

In Figure 3, we present the AUPR results across four datasets. In the CA-Grqc dataset, our proposed measure $SAC_{CC}$ outperforms all the baselines. In the Facebook-large dataset, $SAC_{CC}$ shows strong performance, while PA emerges as the worst performing measure for both the CA-Grqc and Facebook-large datasets. In the web-polblogs dataset, $SAC_{D}$ performs the best among all measures. In the bio-celegans dataset, $SAC_{CC}$ performs better, whereas JC does not performing well on both web-polblogs and bio-celegans. Specifically, for CA-Grqc, our proposed measure $SAC_{CC}$ consistently outperforms RA by 19%, and CCPA, the latest measure, by 28%. For the Facebook-large dataset, the proposed $SAC_{CC}$ demonstrates a 29% enhancement compared to CN, and a significant 46% improvement over CCPA. In web-polblogs, $SAC_{D}$, outpaces CN by 21%, and surpasses CCPA, by 13%. Finally, for bio-celegans, $SAC_{CC}$ excels with a 31% improvement over RA and a notable 37% improvement over CCPA.

### 7.2. Comparing Proposed Measures

**Figure 4:**
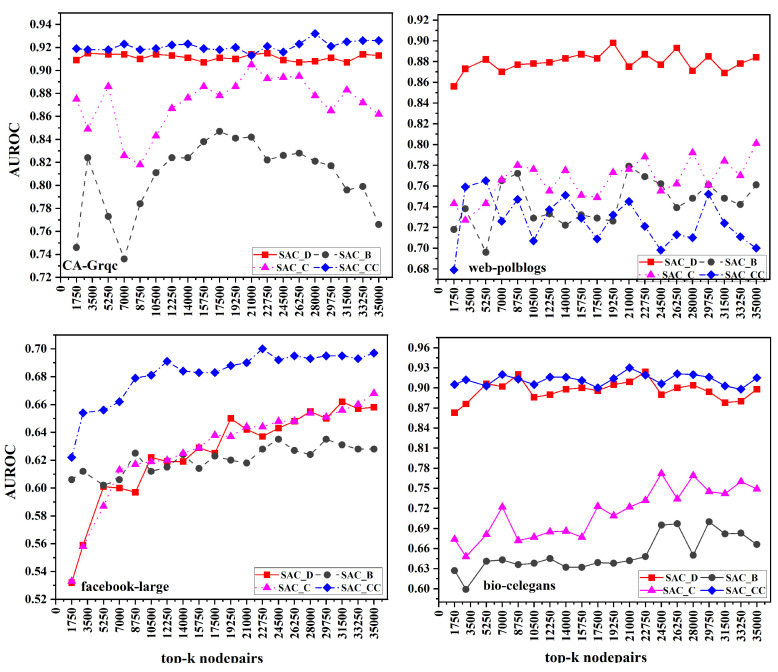
AUROC scores for proposed measures of top 35,000 node pairs across four datasets with SAC_D_ (Similarity based on Average Degree), SAC_B_ (Similarity based on Average Betweenness), SAC_C_ (Similarity based on Average Closeness), and SAC_CC_ (Similarity based on Average Clustering Coefficient).

**Figure 5:**
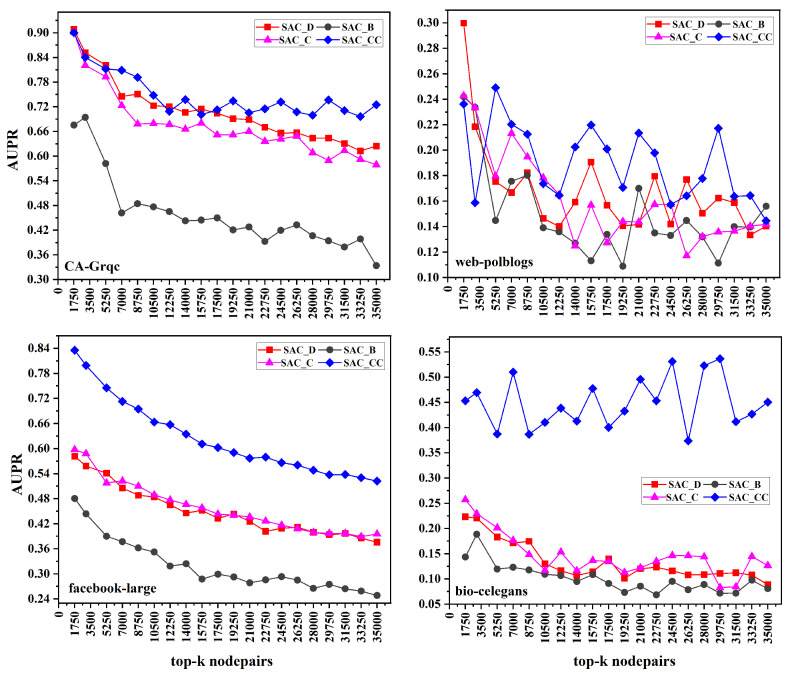
AUPR for proposed measures of top 35,000 node pairs across four datasets with SAC_D_ (Similarity based on Average Degree), SAC_B_ (Similarity based on Average Betweenness), SAC_C_ (Similarity based on Average Closeness), and SAC_CC_ (Similarity based on Average Clustering Coefficient).

In this section, we present a comprehensive comparison study of the suggested similarity measures on a variety of real-world datasets, including web-polblogs, bio-celegans, Facebook-large, and CA-Grqc. The similarity measures we considered were $SAC_{D}$, $SAC_{B}$, $SAC_{C}$, and $SAC_{CC}$. Our results in Figure 4 show that $SAC_{CC}$ consistently performs better in terms of AUROC throughout the networks of CA-Grqc, Facebook-large, and bio-celegans. $SAC_{D}$, however, exhibits the best performance on the web-polblogs dataset. On the other hand, $SAC_{B}$ performs the worst on the CA-Grqc, bio-celegans, and Facebook-large datasets. However, $SAC_{CC}$ performs poorly on the web-polblogs dataset. The web-polblogs dataset pertains to political blogs, where individuals often share their personal experiences rather than consistently citing external sources. The diversity in content within political blogs may contribute to a lower clustering coefficient, leading to the weak performance of $SAC_{CC}$ when compared to $SAC_{D}$, which emphasizes node connectivity over clustering tendencies. When considering the AUPR in Figure 5, $SAC_{CC}$ consistently demonstrates superior performance across all datasets. Conversely, $SAC_{B}$ consistently performs the worst among all measures across all datasets.These results emphasize the influence of a network’s structure and properties on the effectiveness of local similarities based on local and global centralities. Furthermore, it is worth noting that, in various network scenarios, local centralities perform better than global centralities.

### 7.3. Comparing Proposed Measures with Recent Methods like CCPA and KNLP

In Table 4 and Table 5, we randomly chose a few node pairs instead of representing them all. These tables summarize the results based on AUC and AUPR obtained for the proposed algorithms, comparing them with the recent methods CCPA and KNLP on four datasets. It should be noted that we considered top k node pairs, with k=20 datapoints ranging from 1750 to 35,000 i.e., $k=\left\{1750,3500,\ldots ,35,000\right\}$. In Table 4, we examine the Facebook-large dataset with k = 26,250. The AUROC score for the KNLP measure is 0.257. This implies that the KNLP approach encountered difficulties in accurately discerning between positive and negative predictions of link formation in this dataset and under these parameter conditions. The result suggests a higher prevalence of false positives compared to true positives across different datapoint settings, leading to the AUROC value of 0.257. In Table 4, for CA-Grqc dataset, for the top 8750 node pairs, our approach $SAC_{CC}$ outperform the latest measures, CCPA and KNLP, by 6% and 57%. For the top 26,250 node pairs, $SAC_{CC}$ demonstrated significant improvement over CCPA by 7% and over KNLP by 44%. For the Facebook-large dataset, for the top 8750 node pairs, $SAC_{CC}$ excels with an 11% improvement over CCPA and 37% improvement over KNLP. Furthermore, for the top 26,250 node pairs, $SAC_{CC}$ performs best over CCPA and KNLP by 10% and 44%. In web-polblogs, $SAC_{D}$ performs best over CCPA by 11% on the top 8750 and 26,250 node pairs, and also performs best over KNLP by 14% and 12% for the top 8750 and 26,250 node pairs. For bio-celegans, for the top 8750 node pairs, $SAC_{D}$ demonstrates a 10% enhancement compared to CCPA, and a significant 14% improvement over KNLP. Furthermore, for the top 26,250 node pairs, the $SAC_{CC}$ measure outpaces CCPA by 11%, and surpasses KNLP by 12%.

In the context of Table 5, our $SAC_{D}$ approach exhibits superior performance on the CA-Grqc dataset. Specifically, for the top 8750 node pairs, it outperforms the latest measures, CCPA and KNLP, by 37% and 91%, respectively. Additionally, for the top 26,250 node pairs, $SAC_{CC}$ demonstrates a significant improvement over CCPA, showing a 17% advantage, and over KNLP, showcasing a remarkable 70% improvement. Turning to the Facebook-large dataset, $SAC_{CC}$ excels for both the top 8750 and top 26,250 node pairs, surpassing CCPA by 48% and 32%, and outperforming KNLP by 69% and 56%, respectively.

In the case of the web-polblogs dataset, $SAC_{CC}$ outperforms CCPA by 14% and KNLP by 21% for the top 8750 node pairs. Moreover, for the top 26,250 node pairs, $SAC_{D}$ demonstrates a significant improvement over CCPA by 10% and KNLP by 17%. For the bio-celegans dataset, $SAC_{CC}$ showcases a notable 29% enhancement over CCPA and a substantial 36% improvement over KNLP for the top 8750 node pairs. Similarly, for the top 26,250 node pairs, $SAC_{CC}$ outpaces CCPA by 28% and surpasses KNLP by 34%.

### 7.4. Discussion

The experimental result shows that our proposed similarity-based centralities (SAC) measures outperformed state-of-the-art models, when compared with existing local similarity-based link prediction measures and the latest measures, particularly $SAC_{CC}$, outperform existing link prediction measures like JC and KNLP, in terms of AUROC on all datasets. However, $SAC_{CC}$ consistently achieved higher scores in terms of AUPR, indicating its superior predictive power over PA, JC, and KNLP measures on overall datasets. For example, when considering the JC measure applied to the web-polblogs dataset, which represents a network of political blogs, the presence of distinct communities or tightly-connected groups within the network may result in fewer shared connections between nodes from different communities. This phenomenon can lead to less accurate predictions. Moreover, in political blog networks, the formation of links in the preferential attachment (PA) model may depend more on the relevance of topics rather than solely on the connectivity of highly linked political blogs. Consequently, this could lead to lower predictive accuracy compared to models like $SAC_{CC}$ and $SAC_{D}$, which take into account the presence of closely connected communities in the network.

When comparing the proposed measures themselves, our proposed measure $SAC_{CC}$ performed exceptionally well on datasets like CA-Grqc, Facebook-large, and bio-celegans, as it effectively captured the patterns and structures specific to these networks. $SAC_{D}$ performed better on the web-polblogs dataset, where the number of neighbors is crucial for link prediction. However, both $SAC_{B}$ and $SAC_{C}$ exhibited lower levels of information flow between proteins and are less closely connected. Consequently, they achieved lower accuracy compared to $SAC_{CC}$ and $SAC_{D}$. In terms of AUPR, $SAC_{CC}$ consistently outperformed other measures, while $SAC_{B}$ performed the worst for all datasets. This indicates AUPR effectiveness in identifying true positive links while minimizing false positives.

These findings emphasize the importance of considering network structure and properties when selecting the most suitable similarity measures for link prediction.

## 8. Conclusions

In conclusion, our research addresses the challenging task of predicting missing links based on centralties in complex networks. We propose novel similarity measures that incorporate generalized centrality measures, including degree, betweenness, closeness, and clustering coefficient. Our approach identifies top similarity scores by considering the top 20 node pairs. The results, as measured by AUC and AUPR, demonstrate the superior effectiveness of our approach. Our findings highlight the effectiveness of the proposed measures, particularly in the realm of local similarity based on local centrality measures rather than global centralities.

Future research endeavors could extend this work to predicting links using global similarity measures based on global centralities within complex networks. Additionally, we aim to explore similarity-based centralities in hypergraphs as an extension beyond traditional graphs. Furthermore, considering the significance of weighted networks, where edges are assigned different weights to denote the strength or importance of connections between nodes, it would be valuable to explore how the SAC approach performs in such networks, as the weights may influence the centrality measures and, consequently, the similarity scores. Directed networks, where edges have a specific direction, introduce additional complexities in measuring centrality. However, our current focus remains on unweighted, undirected graphs and we intend to explore weighted, directed graphs in future extensions of our work.

## Figures and Tables

**Table 1:** $SAC_{C}\left(v,u\right)$ is the proposed centrality, where C stands for D (degree), B (betweenness), C (closeness), and CC (clustering coefficient).

S.No.	Centrality C	Avg C	${\mathrm{SAC}}_{C}\left(v,u\right)$
1	$C_{D}\left(v\right)=\frac{d_{v}}{n-1}$	$AD\left(G\right)=\frac{\sum_{v\in V(G)}C_{D}\left(v\right)}{N}$	$\begin{array}{c} SAC_{D}\left(v,u\right)=|\{x|x\in \\ \Gamma \left(v\right)\cap \Gamma \left(u\right)\;\mathrm{and}\;D\left(x\right)\geq AD\left(G\right)\}| \end{array}$
2	$C_{B}\left(v\right)=\sum_{p,q\in V}\frac{\sigma_{p,q}\left(v\right)}{\sigma_{p,q}}$	$AB\left(G\right)=\frac{\sum_{v\in V(G)}C_{B}\left(v\right)}{N}$	$\begin{array}{c} SAC_{B}\left(v,u\right)=|\{x|x\in \\ \Gamma \left(v\right)\cap \Gamma \left(u\right)\;\mathrm{and}\;B\left(x\right)\geq AB\left(G\right)\}| \end{array}$
3	$C_{C}\left(v\right)=\frac{n-1}{\sum_{u\neg v}d_{v,u}}$	$AC\left(G\right)=\frac{\sum_{v\in V(G)}C_{C}\left(v\right)}{N}$	$\begin{array}{c} SAC_{C}\left(v,u\right)=|\{x|x\in \\ \Gamma \left(v\right)\cap \Gamma \left(u\right)\;\mathrm{and}\;C\left(x\right)\geq AC\left(G\right)\}| \end{array}$
4	$C_{CC}\left(v\right)=\frac{2K_{v}}{d_{v}\left(d_{v}-1\right)}$	$ACC\left(G\right)=\frac{\sum_{v\in V}C_{CC}\left(v\right)}{N}$	$\begin{array}{c} SAC_{CC}\left(v,u\right)=|\{x|x\in \\ \Gamma \left(v\right)\cap \Gamma \left(u\right)\;\mathrm{and}\;CC\left(x\right)\geq ACC\left(G\right)\}| \end{array}$

**Table 2:** SAC_D_ (Similarity based on Average Degree), SAC_B_ (Similarity based on Average Betweenness), SAC_C_ (Similarity based on Average Closeness), SAC_CC_ (Similarity based on Average Clustering Coefficient), CN (common neighbor), JC (Jaccard coefficient), PA (preferential attachment), RA (resource allocation), AA (Adamic–Adar), CCPA (Common Neighbor and Centrality-based Parameterized Algorithm), and KNLP (keyword network link prediction algorithm) similarity scores for non-adjacent node pairs for a graph are shown in Figure 1.

Various Measures	Node Pair (v,u)	(1,2)	(2,3)	(2,6)	(4,7)	(4,8)	(5,7)
Proposed Measures	**SAC_D_ (v,u)**	1	1	2	2	2	1
	**SAC_B_ (v,u)**	1	1	2	2	1	1
	**SAC_C_ (v,u)**	2	1	2	1	1	1
	**SAC_CC_ (v,u)**	0	0	1	0	2	0
Basic Measures	$S_{v,u}^{CN}$	2	1	2	2	2	2
	$S_{v,u}^{JC}$	0.5	0.2	0.5	0.4	0.4	0.2
	$S_{v,u}^{AA}$	2	0.6	1.3	1.8	1.6	0.9
	$S_{v,u}^{RA}$	0.7	0.2	0.4	0.6	0.5	0.3
	$S_{v,u}^{PA}$	9	6	9	10	10	8
Recent Measures	$S_{v,u}^{CCPA}$	2.4	1.5	2.4	2	2.4	1.5
	$S_{v,u}^{KNLP}$	0.9	0.4	0.7	2.3	0.5	1.2

**Table 3:** Basic properties of datasets.

Datasets	#Nodes	#Edges	#Max. Degree	#Avg. Degree	#Diameter	#Avg. Clust. Coeff.
bio-celegans	453	2025	237	8.94	7	0.646
web-polblogs	643	2280	165	7.09	10	0.232
CA-Grqc	5242	14,496	81	5	17	0.529
Facebook-large	22,470	171,002	709	15.22	15	0.359

**Table 4:** Performance of the proposed measures against existing measures in terms of AUROC for the top *k* predictions, at various thresholds of *k*.

Datasets	*k*	SAC_D_	SAC_B_	SAC_C_	SAC_CC_	CCPA	KNLP
**CA-Grqc**	1750	0.909	0.746	0.875	0.919	0.859	0.549
	8750	0.91	0.784	0.818	0.918	0.859	0.344
	17,500	0.911	0.847	0.878	0.918	0.842	0.392
	26,250	0.907	0.828	0.895	0.923	0.851	0.482
	35,000	0.913	0.766	0.862	0.926	0.853	0.444
**Facebook-large**	1750	0.532	0.606	0.533	0.622	0.626	0.317
	8750	0.597	0.625	0.617	0.679	0.571	0.304
	17,500	0.625	0.623	0.638	0.683	0.607	0.251
	26,250	0.648	0.627	0.648	0.695	0.59	0.257
	35,000	0.658	0.628	0.668	0.697	0.591	0.392
**web-polblogs**	1750	0.856	0.718	0.743	0.679	0.721	0.456
	8750	0.877	0.772	0.78	0.747	0.771	0.384
	17,500	0.883	0.729	0.749	0.709	0.785	0.407
	26,250	0.893	0.739	0.762	0.713	0.788	0.385
	35,000	0.884	0.761	0.801	0.7	0.771	0.376
**bio-celegans**	1750	0.863	0.627	0.674	0.905	0.803	0.79
	8750	0.92	0.636	0.672	0.913	0.822	0.785
	17,500	0.896	0.639	0.723	0.9	0.803	0.824
	26,250	0.9	0.697	0.734	0.921	0.816	0.802
	35,000	0.898	0.666	0.749	0.915	0.832	0.788

**Table 5:** Performance of the proposed measures against existing measures in terms of AUPR for the top *k* predictions, at various thresholds of *k*.

Datasets	*k*	SAC_D_	SAC_B_	SAC_C_	SAC_CC_	CCPA	KNLP
**CA-Grqc**	1750	0.908	0.6756	0.9019	0.9002	0.5403	0.0002
	8750	0.7507	0.4843	0.6783	0.7915	0.5368	0.0002
	17,500	0.7043	0.4498	0.6517	0.7123	0.5104	0.008
	26,250	0.6569	0.4327	0.6487	0.7074	0.5341	0.0023
	35,000	0.6244	0.3336	0.5791	0.7249	0.5345	0.0001
**Facebook-large**	1750	0.5815	0.4803	0.5973	0.8353	0.2132	0.0001
	8750	0.488	0.3619	0.5098	0.6943	0.2092	0.0002
	17,500	0.433	0.299	0.4432	0.6023	0.2151	0.0002
	26,250	0.4115	0.285	0.4081	0.5603	0.2431	0.0001
	35,000	0.3758	0.2482	0.3953	0.5222	0.2268	0.0314
**web-polblogs**	1750	0.2998	0.2419	0.2428	0.2362	0.094	0.003
	8750	0.1822	0.1802	0.1948	0.2126	0.0676	0.0015
	17,500	0.1568	0.1338	0.1273	0.2009	0.0687	0.0024
	26,250	0.1769	0.1447	0.1171	0.1642	0.0733	0.0021
	35,000	0.1403	0.1559	0.1418	0.1446	0.0911	0.0016
**bio-celegans**	1750	0.2232	0.1433	0.2572	0.453	0.0753	0.0211
	8750	0.1744	0.1177	0.1483	0.3867	0.095	0.0273
	17,500	0.1396	0.091	0.1345	0.4003	0.0755	0.0356
	26,250	0.108	0.0786	0.1461	0.3736	0.0921	0.0297
	35,000	0.0887	0.0806	0.1265	0.4505	0.081	0.0265

## Data Availability

No new data were created or analyzed in this study. Data sharing is not applicable to this article.

## Abbreviations

The following abbreviations listed below were used in this paper:

LP	Link prediction
CMs	Centrality measures
CNs	Common neighbors
JC	Jaccard coefficient
AA	Adamic–Adar
RA	Resource allocation
PA	Preferential attachment
D	Degree centrality
B	Betweenness centrality
C	Closeness centrality
CC	Clustering coefficient
CCPA	Common Neighbor and Centrality-based Parameterized Algorithm
KNLP	Keyword network link prediction algorithm
*SAC*_*D*	Similarity based on Average Degree
*SAC*_*B*	Similarity based on Average Betweenness
*SAC*_*C*	Similarity based on Average Closeness
*SAC*_*CC*	Similarity based on Average Clustering Coefficient
AUROC	Area Under the Receiver Operating Characteristic
AUPR	Area Under Precision-Recall
